# Risk Factors and Pain Management in the Incidence of Postoperative Delirium in Elderly Patients: A Retrospective Study

**DOI:** 10.3390/jcm13247624

**Published:** 2024-12-14

**Authors:** Lucas Vizerie, Timothée Morales, Sophie Galey, Franck Montel, Lionel Velly, Nicolas Bruder, Pierre Simeone

**Affiliations:** 1 AP-HM, Department of Anesthesiology and Critical Care Medicine, University Hospital Timone, Aix Marseille University, 13005 Marseille, France; 2 CNRS, Inst Neurosci Timone, Aix Marseille University, UMR7289, 13005 Marseille, France

**Keywords:** postoperative delirium, postoperative pain, pain management, surgery, risks factors

## Abstract

**Background**: Postoperative delirium (POD) is a common surgical complication that increases hospital stay duration, hospitalization costs, readmission rates and mortality. This study aims to describe the incidence of POD in an elderly patient population and to investigate pain assessment as a risk factor for postoperative confusion. Additionally, we aim to determine a predictive model for POD. **Methods**: We conducted a retrospective, observational, single-center study at La Timone Hospital between September 2020 and September 2021. We included patients aged 65 or older, admitted for scheduled or emergency surgical care, with an expected postoperative stay of three days or more and no history of dementia. Data were collected in three stages of hospitalization: preoperative, perioperative, and postoperative. Preoperative data included medical history and lifestyle; perioperative data included surgical and anesthesia characteristics, and postoperative data were collected. **Results**: Of the 531 patients admitted for surgical care, we analyzed 109 patients. Among these, 24 (22%) experienced a POD episode within the first three postoperative days. Age, preoperative cognitive impairments, preoperative sensory deficits, and long-term benzodiazepine use were identified as risk factors for POD. A significant difference in pain levels was also observed for all NRS scores during the first three postoperative days. After multivariate analysis, we retained two predictive models for POD. **Conclusions**: This study identified risk factors for POD and proposed predictive models based on these factors. Two models were particularly notable for their potential use in anesthesia consultations and patient follow-up services to quickly detect patients at risk of POD.

## 1. Introduction

Postoperative delirium (POD) is a common surgical complication that occurs typically between 24 and 72 h postoperatively, without a clear symptom-free interval between surgery and return to the ward. POD is associated with a significant increase in the length of hospital stay, hospital costs, readmission rates, long-term cognitive impairment, and even mortality [1]. In 2011, the total cost of delirium was estimated to be $182 billion annually in Europe [1,2]. Delirium manifests in several clinical forms: hyperactive, hypoactive, and mixed [3,4]. The pathophysiology of delirium remains poorly understood, with main hypotheses currently under investigation [5,6,7]. The incidence of delirium varies widely between studies, ranging from 10% to 70 [8,9]. This variability is explained by the fact that the data in the different studies are not homogeneous, such as the diagnostic criteria for delirium, the population, and the type of surgery studied [10]. The diagnostic reference for delirium screening is the confusion assessment method (CAM), which has a sensitivity of 94% and specificity of 89%, as well as excellent reproducibility. It has been translated and validated in 12 languages, including French [11,12,13].

The pathophysiology of delirium remains poorly understood, with three main hypotheses currently discussed, focusing on cholinergic deficit and dopaminergic excess conditions: (i) Imbalance in intracerebral neurotransmission favors cholinergic deficit and dopaminergic excess conditions. Acetylcholine, crucial for cognitive activities, is affected, and dopaminergic drugs can induce confusion [5]. (ii) The involvement of cytokines like interleukins (IL) IL-1, IL-2, IL-6, tumor necrosis factor, and interferon gamma increases blood–brain barrier permeability and disrupts neurotransmission [6]. (iii) The activation of the sympathetic nervous system and the hypothalamic–pituitary–adrenal axis due to stress from illnesses or trauma increases circulating cytokines and hypercortisolism. This state is associated with serotoninergic receptor dysfunction (5-HT1A) [14]. Elderly patients are more affected due to known risk factors for postoperative delirium, including cognitive impairment, sensory deficits, comorbidities, and malnutrition [15,16,17]. Numerous studies have identified preoperative and intraoperative risk factors for POD, including comorbidities, preoperative cognitive impairment, depression, alcohol consumption, and the use of anticholinergic medications [15,18,19,20]. Additionally, surgical risk factors include surgical specialty (with visceral, orthopedic, and cardiothoracic surgeries being the most significant), intraoperative bleeding, and the duration of surgery. Elderly patients are at higher risk of developing POD due to increased exposure to known risk factors such as cognitive impairment, sensory deficits, and polypharmacy. Few published studies have focused on the incidence of POD and its association with modifiable risk factors such as pain and analgesic management, with no consensus on their conclusions [2,3,21]. The objective of this study was to describe the incidence of POD in an elderly patient population and to investigate pain assessment as a risk factor for postoperative confusion. The secondary objective was to determine a predictive model for POD based on these data.

## 2. Materials and Methods

### 2.1. Patient Recruitment

We conducted this retrospective single-center study between September 2020 and September 2021 to investigate the association between postoperative pain and POD. This protocol was validated by the Delegation for Clinical Research and Innovation (DRCI) of the AP-HM. Ethical approval for this study was not required because it was a retrospective study, and DRCI approved the use of the data (PADS20-219).

We included patients aged 65 years or older, admitted to La Timone Hospital in Marseille for scheduled or urgent surgical management, with an expected postoperative length of stay greater than or equal to 3 days. We chose to include patients older than 65 years because they are the most at risk of developing an episode of POD. All included patients were fluent in French, had no history of dementia, and underwent surgery in the fields of neurosurgery, visceral surgery, or orthopedic surgery.

We collected our data during three stages of their hospitalization: the preoperative period by collecting their history and lifestyle, the intraoperative period by recording the characteristics of the surgery and the anesthesia, and the postoperative period by making a daily visit to the department during three consecutive days following their surgery as routine care in our ward.

### 2.2. Preoperative Data Collection

According to previous studies [18,19,22,23,24,25], the following were collected: age, sex, history of stroke, epilepsy, the presence of auditory or visual sensory deficit, BMI, personal history, and alcohol and tobacco consumption. The severity of comorbidities was measured by the Charlson comorbidity score [26], as well as by the ASA score, or physical status score.

Preoperative pain and its evolution postoperatively were recorded using a numeric rating scale (NRS) from 0 to 10. We also recorded the worst NRS during the week preceding surgery (worst NRS for 7 days). Long-term preoperative treatment with benzodiazepine, morphine, tricyclic antidepressant, antipsychotic, and antiepileptic drugs, which cause postoperative confusion, was collected [22]. The presence of polymedication (>4 drug classes) was also specified.

In addition, the presence of chronic preoperative depression [24,25] was reported using a simple diagnostic test validated in French, the mini GDS test [27]. We have also carried out a CODEX (cognitive disorders examination) test [28] to detect patients with cognitive disorders preoperatively. Its main advantage is that it is simple and rapid (<3 min) in the evaluation of memory and other cognitive functions. It constitutes a screening tool of assigning a very low (A) low (B) high (C) or very high (D) probability of the presence of cognitive frailty. At the biological level, preoperative hemoglobin, albumin, and creatinine were collected.

### 2.3. Intraoperative Data Collection

These data were collected in real time during anesthesia by the operating room team (resident, nurse, or anesthesiologist) in charge of the patients. We reported surgical specialty as orthopedic, visceral, or neurological surgery. For each surgical specialty, we classified the procedures as major or intermediate surgery. Within neurosurgery, we specified brain surgery from other types.

In addition, the urgent or scheduled nature of the surgery, its date with a notion of seasonality, the type of anesthesia (only regional anesthesia (RA); general anesthesia (GA); and general and regional analgesia (GRA)), the presence of intraoperative hypotension (stratified every 5 min) [29], and transfusion [30] were analyzed to determine the intraoperative risk factors for POD [23]. The anesthesia protocols were detailed for patients who received peripheral or central regional anesthesia. For patients managed with GA, the dosage of anesthetic drugs (converted into dose/weight) was collected. We analyzed the intraoperative drugs most likely to cause delirium: morphine, ketamine [31,32,33], and analgesics such as nefopam and tramadol [34].

### 2.4. Measurement of Pain and Analgesic Intake in the Postoperative Period

The main objective of our study was to assess the association between pain in the three postoperative days and the incidence of POD. The nurses of the surgical departments notified, with the help of the professional prescription software used, the NRS of the patients present in their unit three times a day (morning, noon, and evening). In addition, when visiting the surgical wards to assess the presence of POD, our team also performed an NRS with the included patients on a daily basis [35,36,37]. Attention was paid to the change in NRS between the intraoperative period and postoperative period. A difference of 3 points or more in NRS was considered significant [24]. Analgesic intake in the three days following surgery was recorded using the same software, notifying each daily analgesic intake as well as their dosage.

The use of the different analgesics was recorded and also expressed according to the levels recommended by the World Health Organization (1, 2, or 3). These dosages were also expressed in dose/weight. Particular attention was paid to the use of nefopam, tramadol [13], and morphine derivatives (PerOs (PO) or IV) in order to analyze their involvement in the development of POD.

### 2.5. Diagnosis and Detection of Delirium

As routine standard care, during the first three days postoperatively (Day 1 defined as the day after surgery), a member of the anesthesia team visited the included patients daily in the intensive care unit or in the conventional surgical ward. POD was diagnosed using the CAM criteria with four main criteria: a change in mental status, inattention, disorganized thinking, and fluctuating consciousness [11]. The personnel in charge of this questionnaire were trained to perform this test in the presence of the investigator. The clinical form of delirium was evaluated as hypoactive, hyperactive, or mixed [3,15]. We studied the time between surgery and the onset of delirium, as well as its duration. We also looked for classical somatic causes that can occur in confused patients such as fecal impaction, acute urine retention, dysnatremia, or morphine overdose. We systematically looked for these somatic etiologies in each confused patient using the confusion assessment method (Supplementary Material—Confusion Assessment Method (CAM)).

### 2.6. Patient Follow-Up and Complications

In the event of POD, the methods of management within the surgical departments, whether medical or physical, were collected, including in particular the presence of the family [38]. At the La Timone hospital, a mobile geriatric team offers management when such an episode appears. Their request by the surgical teams was also specified. We studied the length of hospitalization [21] of each included patient. Finally, we collected postoperative complications during the entire hospital stay.

### 2.7. Statistical Analysis

The data were tested for normality of distribution (Shapiro–Wilk test) and are presented as means and standard deviations, for continuous variables or medians, and inter-quartile ranges, for non-continuous variables, according to their distribution. The categorical variables are presented as *n* (%). Comparisons between groups according to their results and developments were made using Fisher’s exact test, or Student’s *t*-test, or Wilcoxon test, according to their distribution. Multivariate analyses were performed by linear regression. The Pearson’s square correlation (R2) was used to evaluate the correlation of the different variables. All analyses were performed using JMP version 13. Comparisons between ROC curves were performed with MedCalc version 11. Missing data were not replaced. The significance level of *p*-value was set at 0.05. Multiple comparisons were corrected by taking into account a false discovery rate (FDR) correction. The analysis was performed in a blinded condition.

## 3. Results

### 3.1. Population Characteristics

**Figure 1:**
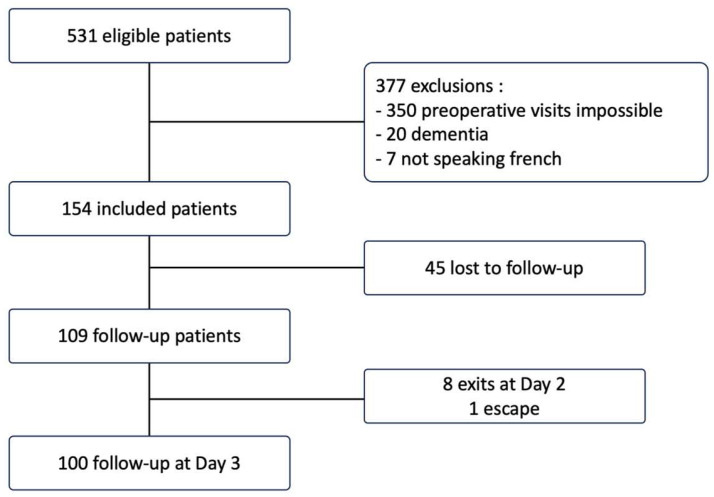
Flow chart of the study.

From September 2020 to September 2021, 531 patients were admitted to La Timone Hospital for surgical management. Of these, 154 met the eligibility criteria. Ultimately, 109 patients were analyzed, with 45 patients lost to follow-up. Nine patients were lost to follow-up by day 3. Figure 1 illustrates the study’s follow-up chart.

The demographic characteristics of the patients and their preoperative cognitive status are presented in Table 1.

Of these 109 patients, 24 (22%) experienced an episode of postoperative delirium (POD) during the first 3 postoperative days. The median age of the included patients was 75 [70–81.5] years. Patients with POD were significantly older (74 [68–77.5] vs. 82 [73–86.5] years; *p* = 0.002). There was no significant gender difference between the two populations (male: 43 (50.6%) vs. 7 (29.1%); *p* = 0.051).

Twenty-two percent of the study population had a preoperative sensory deficit, primarily auditory (90%). Preoperative sensory deficit was more frequent in patients with POD (13 (15.3%) vs. 11 (45.8%); *p* = 0.002). Fifteen patients (13.8%) in our cohort had preoperative cognitive frailty, which was more frequent in patients with POD (C/D Codex test: 6 (7.1%) vs. 9 (37.5%); *p* < 0.001). In our population, 46.8% of patients were treated daily with more than four drug classes, defined as polypharmacy. Polypharmacy was also more frequent in patients with POD (35 (41.2%) vs. 16 (66.7%); *p* = 0.027).

Regarding lifestyle habits, 16.5% of the included patients consumed at least one glass of alcohol per day, 15.6% used benzodiazepines, 4.6% used tricyclic antidepressants, and 4.6% used morphine. Long-term use of benzodiazepines (8 (9.4%) vs. 9 (37.5%); *p* < 0.001) and tricyclic antidepressants (2 (2.35%) vs. 3 (12.5%); *p* = 0.036) was more frequent in patients with POD. There was no significant difference in depressive disorder proportion between the two groups (26 (30.6%) vs. 10 (41.7%); *p* = 0.534).

### 3.2. Characteristics of Surgery and Anesthesia

The characteristics of the surgery and anesthetic management are reported in Table 2. Eleven patients (10.1%) were managed in emergency conditions, with no significant difference between the two groups (7 (8.2%) vs. 4 (16.7%); *p* = 0.199). Intracranial surgeries concerned 14 patients (12.8%) in our population, with no increased incidence of POD for these patients (10 (11.8%) vs. 4 (16.7%); *p* = 0.526).

Concerning anesthetic management, 44 (40.4%) patients underwent general anesthesia (GA), 62 (56.9%) underwent general and regional anesthesia (GRA), and 3 (2.7%) patients underwent regional anesthesia (RA) alone. The type of anesthesia did not influence the incidence of POD (*p* = 0.436). When anesthesia combined general and regional techniques, the regional technique was central in 37% and peripheral in 63% of cases.

In our cohort, 35.8% of patients experienced intraoperative hypotension. The duration of hypotension was 5–10 min for 22% of patients, 10–15 min for 6.4%, and over 15 min for 7.4% of them. Patients with POD had a greater duration of intraoperative hypotension (3 [3–7] vs. 10 [3–15] minutes; *p* = 0.003). The anesthetic agents and intraoperative treatments used are presented in Supplementary Table S1. There was no significant difference in treatment used between the two groups regarding the incidence of POD.

### 3.3. Pain and Postoperative Analgesic Management

Preoperative pain, assessed by the numerical rating scale (NRS), was 2 [0–4]. The worst NRS during the week before surgery was higher in patients with POD (2 [0–5] vs. 6 [0–8]; *p* = 0.038). Two patients (1.8%) were premedicated the day before surgery.

**Figure 2:**
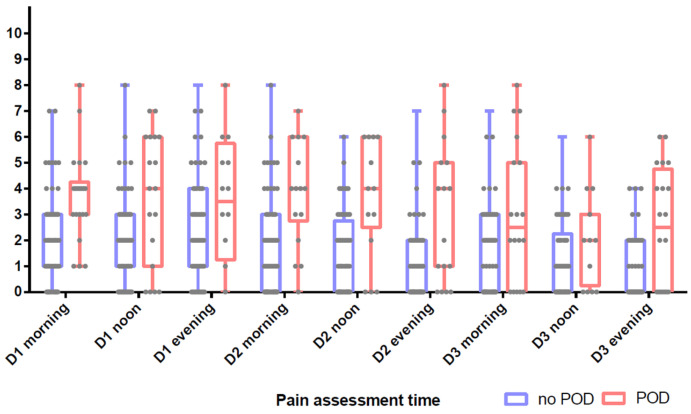
Postoperative pain assessment with numeric rating scale between no POD and POD patients (Wilcoxon test).

Data concerning the evaluation of preoperative and postoperative pain are reported in Figure 2. Confused patients experienced more postoperative pain than non-confused patients. NRS on the morning after surgery (NRS Day 1 morning) was greater in patients with POD (2 [1–3] vs. 4 [3,4]; *p* < 0.001). This significant difference was observed for all NRS collected during the first 3 postoperative days.

Analgesic management during the first three postoperative days in the ward is reported in Supplementary Table S2. During the first three postoperative days, nine (8.3%), seventeen (15.7%), and fourteen (14%) patients received oral morphine, and fourteen (12.8%), five (4.6%), and five (5%) patients received intravenous (IV) morphine. Confused patients received tramadol more frequently on Day 1 (9 (37.5%) vs. 24 (22%); *p* = 0.040). Nefopam use was not associated with an increase in POD. Additionally, the use of IV morphine on Day 1 and Day 3 was more frequent in patients with POD (Day 1: 7 (29.2%) vs. 14 (12.8%); *p* = 0.013) (Day 3: 5 (5%) vs. 3 (13%); *p* = 0.048). This variation in frequency was not found with oral morphine intake at Day 1 and Day 3, respectively.

### 3.4. Types, Etiologies, Complications, and Management of POD

POD was observed mainly during the first two days after surgery. Seventeen patients (70.8%) became confused on the first postoperative day, five patients (20.8%) on Day 2, and two patients (8.3%) on Day 3. The prevalence of POD and its type are transcribed in Supplementary Table S3. Five patients (20.8%) had a diagnosed somatic cause, where three had acute urinary retention and two had fecal impaction. A medical etiology was found in three patients (12.5%) with delirium, where one had hemorrhagic remodeling after brain surgery, one had hydrocephaly after brain surgery, and one had a morphine overdose and hyponatremia.

Regarding the management of POD episodes, mechanical restraint was prescribed for six patients (33.3%), benzodiazepines for ten patients (41.7%), and antipsychotics for four patients (16.7%). Among the confused patients, family visits during the postoperative stay were authorized for four patients (16.7%). The duration of POD was 1 [1–2] day. The length of hospital stay was greater in confused patients (7 [5–10] vs. 10 [7–17] days; *p* < 0.001). Evaluation by a geriatric team was requested for three confused patients.

The average duration of POD in these patients was 2 days. A return to the operating room was necessary in three (12.5%) of the confused patients and four (4.7%) of the non-confused patients (*p* = 0.169). One patient died during hospitalization with postoperative confusion. The etiology of death was not clearly established, although pulmonary embolism appeared to be the most likely cause.

### 3.5. Multivariate Analyses and Predictive Models

**Figure 3:**
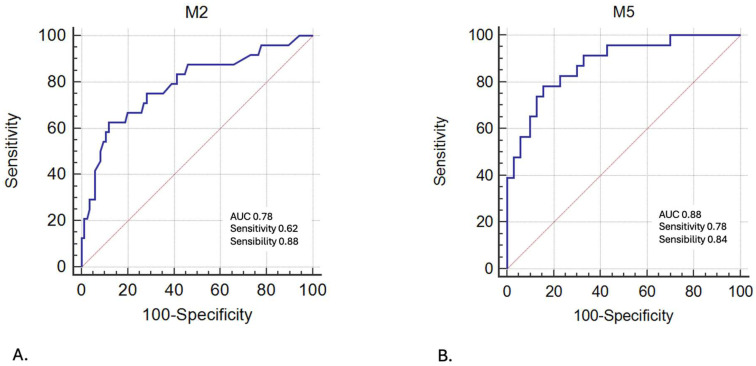
Predictive models of postoperative delirium with Model 2 (**A**) and Model 5 (**B**).

We performed multivariate analyses by integrating preoperative, intraoperative, and postoperative variables showing a significant difference in the univariate analysis. Several analyses were conducted to develop the best-performing and most clinically relevant predictive model for postoperative confusion. The multivariate analyses are presented in Supplementary Table S4 with the criteria considered in the different models. The predictive models are reported in Figure 3, with a comparison of these models in Supplementary Figure S1, allowing us to extract two main models of clinical interest:

- Model 2 (Se = 62.5%, Sp = 88.2%; AUC = 0.79) incorporates a pre-anesthetic assessment with patient demographics and clinical data;
- Model 5 (Se = 78.3%, Sp = 84.3%; AUC = 0.88) excludes preoperative assessments and incorporates intraoperative data and pain assessment at Day 1.

## 4. Discussion

Our study shows that postoperative pain was higher in patients with POD, highlighting the potential importance of early diagnosis and treatment of this pain. Our study population is comparable to previous studies regarding age, preoperative treatments, prevalence of cognitive disorders, and depression.

Twenty-four (22%) patients presented with an episode of POD during the first three postoperative days. In other studies dealing with POD, this incidence is extremely variable, ranging from 10% to 70%. A recent meta-analysis including twenty-two studies found an incidence of POD similar to ours at 17.6% [39].

In our study, there was no statistically significant difference regarding the incidence of POD when looking at the types of anesthesia and anesthetic agents used. This result is comparable to the data in the literature; the meta-analysis by Mason et al. [40] in 2010 found that general, regional, or a combination of these two methods did not correlate with an increase in POD.

Severe pain in the postoperative period rarely exceeds 72 h [41]. The evaluation and treatment of postoperative pain are markers of the quality of care within institutions. In addition, postoperative pain control can have a significant impact on patient recovery, postoperative morbidity, and length of hospital stay. Confused patients had more postoperative pain than patients without POD, whether at Day 1, Day 2, or Day 3. In the studies of Lynch et al. [36] in 1998 and Xue et al. [42] in 2016, patients with POD had proportionally more tramadol at Day 1 and opioid at Day 1 and Day 3. Regarding postoperative analgesic management, Vaurio et al. [43] showed that oral morphinics decreased the risk of POD compared to intravenous (IV) morphinics. Morrison et al. found that the incidence of POD increased for morphine dosages < 10 mg/d, and on the contrary, daily dosages greater than 10 mg seemed to be a protective factor [35]. Finally, a recent 2018 study [44] claimed that opioid use in the perioperative period was associated with increased POD. In our study, the administration of IV morphine seems to increase the incidence of POD, but no statistical analysis of the dosages could be performed because of the small number of patients concerned. However, a recent study coordinated by the SFAR suggested that confusion would be declared as a side effect of morphine in less than 1% of cases, implying that the iatrogenic linked to the use of analgesics would be secondary to the potential risk factor that pain may favor the occurrence of POD [45]. Regarding ketamine, there were no confused patients in patients who received ketamine at anesthetic induction. A recent, prospective, randomized, two-group study with ketamine versus placebo published in *The Lancet* in 2017 [32] concluded the same results.

Concerning the preoperative risk factors for POD, our results are comparable to those of previous studies on the subject [15,44,46,47]. In our study, the risk factors for POD included age, preoperative cognitive impairment, the presence of a preoperative sensory deficit, benzodiazepine and tricyclic medication, and the worst NRS prior to surgical management.

In our study, among the intraoperative data collected, only hypotension shows an association with the incidence of POD, as shown in the prospective study of Wang et al. [30] and Jiang et al. [48], even if other older studies were not consistent [29,49].

This work investigating POD has several strengths. First, unlike most studies of POD and its relationship to postoperative pain that have included only orthopedic surgeries [35,37,39], our study included other surgical specialties such as visceral surgery and neurosurgery. In addition, this study is one of the first to focus on POD by including intracranial surgery. It should be noted that in our cohort, patients undergoing brain surgery were not more confused than those undergoing other surgeries. A study in 2006 in the *Journal of Korean Neurosurgical* also found no link between brain surgery and POD [41]. Secondly, contrary to other studies dealing with POD, we were interested not only in the preoperative factors predisposing to POD but also in the intraoperative factors by including surgical and anesthetic management as well as the postoperative factors by considering the study of postoperative pain. The presence of a sensory deficit is a proven risk factor for delirium outside of surgical management [50]. However, its presence has never been studied in studies of POD. Our study is, to our knowledge, the first to include a search for a preoperative sensory deficit in POD. This may thus potentially open a reflection on factors that can be modified during the management of patients to reduce the incidence of POD and thus constitutes a potential pathway for the prevention of POD. Third, we performed several predictive models of POD based on the statistically significant factors in the univariate analysis. We chose to include morning NRS on Day 1 of surgery in our predictive model for POD because of its representative nature of pain experienced during the first night after surgery. Model 2 showed good specificity (88.2%) while its sensitivity is lower than the other models (62.5%). It could allow us to anticipate an episode of POD in a patient presenting during anesthesia consultation and meeting these criteria. In view of the statistical analyses, the performance of Model 5 is better than Model 2. We can suggest the use of Model 5 in cases where the patient will be followed during the postoperative period by a team that will diagnose and treat postoperative pain. In cases where patients are not followed up or are only minimally followed up during the postoperative period, the use of Model 2 seems more appropriate. These models therefore require work with a prospective validation cohort to be transposed into clinical practice.

Our work also has several limitations. Indeed, few patients have been operated on RA alone. It is known that the risk of intraoperative hypotension during this type of anesthesia decreases compared to GA. These patients are operated on for simple vascular or orthopedic surgeries, with a predicted postoperative length of stay of less than 3 days, making their inclusion in our cohort incompatible. Thus, it is possible that this type of patient has different POD characteristics from those we described. Similarly, we used the NRS to assess postoperative pain. The NRS is a self-report pain scale. A hetero-report pain scale, such as the DOLOPLUS-2 scale, might have seemed more appropriate for assessing pain in confused patients. However, in all previous robust studies dealing with postoperative pain and confusion, self-report scales have been used [24,35,36,43]. Similarly, our data collection did not exhaustively consider blood loss, although this factor is known to favor the occurrence of POD. Finally, concerning the study of intraoperative hypotension, we defined it in patients with a MBP < 65 mmHg. Among previous studies dealing with hypotension and the incidence of POD, there is no consensus on the definition of hypotension. For example, Marcantonio et al. [49] defined intraoperative hypotension as a decrease in more than 66% in systolic or decrease in SBP < 90 mmHg, whereas Hirsch et al. in 2015 [29] defined hypotension as a decrease of 20, 30, or 40% in SBP or MBP < 50 mmHg. Thus, it is possible that these different definitions of intraoperative hypotension make it difficult to extrapolate the results and also induce a classification bias for this type of event.

## 5. Conclusions

By studying our cohort of elderly patients preoperatively and postoperatively, we were able to characterize the existing clinical differences between patients with and without POD. Subsequently, this analysis allowed us to identify risk factors for POD. This study highlights a relationship between pain and POD and underscore the importance of prevention, early diagnosis, and treatment for postoperative pain. Based on these risk factors, we were able to propose predictive models of POD, including two models that could potentially be used during the anesthesia consultation and during the follow-up of patients in the wards to rapidly detect patients at risk of POD. This work opens the opportunity for further studies to validate these predictive models.

## Figures and Tables

**Table 1:** Demographic characteristics of the general population, absence of postoperative delirium (POD), and POD patients.

	Study Population	Absence POD	POD	*p* Value
	*n* = 109	*n* = 85	*n* = 24	
Age (years)	75 [70–81.5]	74 [68–77.5]	82 [73–86.5]	0.002
Age < 70 years *n* (%)	26 (23.8%)	24 (28.2%)	2 (8.3%)	0.043
Gender, male *n* (%)	50 (45.6%)	43 (50.6%)	7 (29.1%)	0.050
Weight (kg)	68.5 [59.5–80]	69.5 [61–80]	61.5 [56–71.5]	0.063
BMI (kg/m^2^)	25.2 [24.4–26.1]	25.5 [24.6–26.4]	24.3 [22.2–26.4]	0.140
ASA Score	2 [2–3]	2 [2–3]	3 [2–3]	0.115
Season *n* (%)				0.813
Winter	43 (47.7%)	41 (48.2%)	11 (45.8%)	
Spring	14 (12.8%)	12 (14.1%)	2 (16.7%)	
Summer	11 (10.1%)	9 (10.6%)	2 (16.7%)	
Autumn	32 (29.3%)	23 (27.1%)	9 (37.5%)	
Charlson score	5 [4–6]	5 [4–6]	5 [4–7]	0.213
Preoperative sensory deficit *n* (%)	24 (22.0%)	13 (15.3%)	11 (45.8%)	0.003
preoperative alcool consumption *n* (%)				0.403
Absence	91 (83.5%)	70 (82.3%)	21 (87.5%)	
1 glass/d	12 (11.0%)	9 (10.6%)	3 (12.5%)	
≥2 glasses/d	6 (5.5%)	6 (7.1%)	0 (0.0%)	
Active smoking (%)	10 (9.2%)	8 (9.4%)	2 (8.3%)	0.87
Preoperative treatment *n* (%)				
Benzodiazepine	17 (15.6%)	8 (9.4%)	9 (37.5%)	<0.001
Tricyclic	5 (4.6%)	2 (2.35%)	3 (12.5%)	0.036
Opioid	5 (4.6%)	3 (3.6%)	2 (8.3%)	0.327
Antiepileptic	5 (4.6%)	5 (5.9%)	0 (0%)	0.224
Antipsychotic	2 (1.8%)	1 (1.2%)	1 (4.2%)	0.335
Depression *n* (%)	36 (33.0%)	26 (30.6%)	10 (41.7%)	0.534
Preoperative cognitive frailty (Codex Test) *n* (%)	15 (13.8%)	6 (7.1%)	9 (37.5%)	<0.001
Physical activity *n* (%)	40 (37%)	32 (38.1%)	8 (33.3%)	0.430
Polymedication n (%)	51 (46.8%)	35 (41.2%)	16 (66.7%)	0.027
Creatinine serum (µmol/L)	85.5 [78.3–92.7]	86 [77–94.7]	83 [66.2–96.5]	0.746
Haemoglobine (g/dL)	12.9 [12.6–13.2]	13 [12.7–13.4]	12.8 [11.7–14.6]	0.189
Albumin (g/dL)	42.3 [39.9–44.5]	42.6 [39.9–44.9]	41.1 [38.1–43.3]	0.307
Premedication *n* (%)	2 (1.8%)	2 (2.35%)	0 (0%)	0.448

Data are expressed as median [25th–75th quartile] and number of subjects (%). Comparisons between groups according to their results and developments were made using Fisher’s exact test, or Student’s *t*-test, or Wilcoxon test, according to their distribution.

**Table 2:** Characteristics of surgery and anesthesia.

	Study Population	Absence POD	POD	*p* Value
	*n* = 109	*n* = 85	*n* = 24	
Type of Anesthesia *n* (%)				0.436
General	44 (40.4%)	37 (43.5%)	7 (29.2%)	
General and Regional	62 (56.9%)	46 (54.1%)	16 (666%)	
Regional	3 (2.7%)	2 (2.4%)	1 (4.2%)	
Type of RA *n* (%)				
Perimedullary	20 (18.3%)	15 (17.6%)	5 (20.8%)	0.725
Extra-axiale	44 (40.3%)	33 (38.8%)	11 (48.8%)	0.469
Surgery schedule *n* (%)				
Morning	51 (46.8%)	41 (48.2%)	10 (41.7%)	0.125
Noon	45 (41.3%)	36 (42.4%)	9 (37.5%)	0.127
Evening	13 (11.9%)	8 (9.4%)	5 (20.8%)	0.129
Emergency *n* (%)	11 (10.1%)	7 (8.2%)	4 (16.7%)	0.199
Surgical Specialty *n* (%)				0.387
Neurosurgery	28 (25.7%)	23 (27.1%)	5 (20.8%)	
Visceral Surgery	48 (44%)	39 (45.9%)	9 (37.5%)	
Orthopedic	33 (30.3%)	23 (27.1%)	10 (34.7%)	
Brain Surgery *n* (%)	14 (12.8%)	10 (11.8%)	4 (16.7%)	0.526
Surgery Duration (min)	110 [60–180]	100 [60–150]	120 [60–240]	0.980
Hypotension duration (min)	3 [3–10]	3 [3–7]	10 [3–15]	0.003
Intraoperative hypotension, *n* (%)				
<5 min	70 (64.2%)	59 (69.4%)	11 (45.8%)	0.040
5–10 min	24 (22%)	21 (24.7%)	3 (12.5%)	0.202
10–15 min	7 (6.4%)	2 (2.35%)	5 (20.8%)	0.005
>15 min	8 (7.34%)	3 (3.5%)	5 (20.8%)	0.012
BIS *n* (%)	46 (42.2%)	35 (41.2%)	11 (45.8%)	0.683

Data are expressed as median [25th–75th quartile] and number of subjects (%). Comparisons between groups according to their results and developments were made using Fisher’s exact test, or Student’s *t*-test, or Wilcoxon test, according to their distribution. RA denotes for Rachianalgesia, BIS denotes for Bispectral Index.

## Data Availability

Data is available upon reasonable request.

## Supplementary Materials

The following supporting information can be downloaded at: https://www.mdpi.com/article/10.3390/jcm13247624/s1, Supplementary Table S1: intraoperative and postoperative anesthetic agents; Supplementary Table S2: analgesics used postoperatively at Day 1 (D1), Day 2 (D2), and Day 3 (D3); Supplementary Table S3: prevalence of postoperative delirium; Supplementary Table S4: univariate and multivariate analysis and presentation of different predictive models; Supplementary Figure S1: comparison of areas under the curve.
