# Supplementary material for: Risk Factors and Pain Management in the Incidence of Postoperative Delirium in Elderly Patients: A Retrospective Study
Source: J Clin Med. 2024 Dec 14;13(24):7624. doi: 10.3390/jcm13247624 (PMC11728186; doi:10.3390/jcm13247624)
Supplement: Supplementary file 1 [file jcm-13-07624-s001.zip › jcm-3330328-supplementary.pdf]

Confusion Assessment Method (CAM)

Short form

The diagnosis of delirium by CAM requires the presence of BOTH features ***A*** and ***B***		
**C A M**<br>**Confusion Assessment Method**	**A.**<br>**Acute onset**<br><br>and<br><br>**Fluctuating course**	Is there evidence of an acute change in mental status from patient baseline?<br><br>Does the abnormal behavior:<br>➢ come and go?<br>➢ fluctuate during the day?<br>➢ increase/decrease in severity?
	**B.**<br>**Inattention**	Does the patient:<br>➢ have difficulty focusing attention?<br>➢ become easily distracted?<br>➢ have difficulty keeping track of what is said?
	**AND the presence of EITHER feature *C or D***	
	**C.**<br>**Disorganized thinking**	Is the patient's thinking<br>➢ disorganized<br>➢ incoherent<br>For example does the patient have<br>➢ rambling speech/irrelevant conversation?<br>➢ unpredictable switching of subjects?<br>➢ unclear or illogical flow of ideas?
	**D.**<br>**Altered level of consciousness**	Overall, what is the patient's level of consciousness:<br>➢ alert (normal)<br>➢ vigilant (hyper-alert)<br>➢ lethargic (drowsy but easily roused)<br>➢ stuporous (difficult to rouse)<br>➢ comatose (unrousable)

Adapted with permission from: Inouye SK, vanDyck CH, Alessi CA, Balkin S, Siegal AP, Horwitz RI. Clarifying confusion: The Confusion Assessment Method. A new method for detection of delirium. Ann Intern Med. 1990; 113: 941-948. Confusion Assessment Method: Training Manual and Coding Guide, Copyright © 2003, Hospital Elder Life Program, LLC.

Please see the **CAM Training Manual**, available at
http://www.hospitalelderlifeprogram.org/private/cam-disclaimer.php?pageid=01.08.00

Le diagnostic de l'état confusionnel aigu requiert la présence des critères 1, 2 et 3 ou 4

Supplementary Table S1. Intraoperative and postoperative anesthetic agents

	Study population n=109	No POD n=85	POD n=24	*p Value*
**Intraoperative agents**				
Remifentanil (%)	17 (15.9%)	13 (15.7%)	4 (16.7%)	0.906
Sufentanil	89 (81.6%)	70 (82.3%)	19 (79.2%)	0.722
Sufentanil (dose/weight in µg) treated patients	0.31 [0.24-0.42]	0.27 [0-0.48]	0.3[0.24-0.37]	0.431
Sufentanil (dose/weight in µg)	0.25 [0-0.39]	0.27 [0-0.48]	0.25[0-0.34]	0.326
Ketamine (%)	39 (36.1%)	32 (37.6%)	7 (30.4%)	0.522
Droleptan (%)	71 (65.1%)	55 (64.7%)	16 (66.7%)	0.859
Dexamethasone (%)	77 (70.6%)	61 (71.8%)	16 (66.6%)	0.628
**Post-operative analgesia**				
Acupan n (%)	45 (54.2%)	38 (55.9%)	7 (46.7%)	0.517
Acupan dosage (mg/kg)	0.20 [0-0.3]	0.22 [0-0.30]	0[0-0.29]	0.431
Tramadol n (%)	48 (57.8%)	42 (61.8%)	6 (40%)	0.122

Data are expressed as median [25th-75th quartile] and number of subjects (%).

Supplementary Table S2. Analgesics used postoperatively at day 1 (D1), day 2 (D2), day 3 (D3)

	Study population n=109	No POD n=85	POD n=24	*p Value*
**POST-OPERATIVE D1**				
Paracetamol n (%)	96 (88%)	76 (89.4%)	20 (83.3%)	0.534
Nefopam n (%)	43 (29.8%)	36 (42.9%)	7 (29.2%)	0.227
Tramadol n (%)	24(22%)	15 (19.6%)	9 (37.5%)	***0.04***
D1 Morphine PO n (%)	9 (8.3%)	8 (9.4%)	1 (4.2%)	0.609
D1 Morphine IV n (%)	14 (12.8%)	4 (8.2%)	7 (29.2%)	***0.013***
**POST-OPERATIVE D2**				
Paracetamol n (%)	97 (90.5%)	75 (90.4%)	22 (91.7%)	0.847
Nefopam n (%)	27 (25.2%)	22 (26.5%)	5 (20.8%)	0.573
Tramadol n (%)	0.5 [0.24-0.77]	0.18 [0.44-0.77]	0.57 [0.44-1.28]	0.192
D2 Morphine PO n(%)	17 (15.7%)	13 (15.4%)	4 (16.7%)	0.887
D2 Morphine IV n(%)	5 (4.6%)	3 (3.57%)	2 (8.3%)	0.36
**POST-OPERATIVE D3**				
Paracétamol n(%)	82 (82%)	62 (81.6%)	20 (83.3%)	0.845
Nefopam n (%)	15 (15%)	12 (15.8%)	3 (12.5%)	0.694
Tramadol n (%)	9 (9%)	6 (7.9%)	3 (12.5%)	0.492
D3 Morphine PO n (%)	14 (14%)	10 (13.6%)	4 (16.7%)	0.187
D3 Morphine IV n (%)	5 (5%)	2 (2.8%)	3 (13%)	***0.0479***

Data are expressed as median [25th-75th quartile] and number of subjects (%).

Supplementary Table S3. Prevalence of postoperative delirium

	Study population n=109	POD n=24
Delirium Day1 n (%)	17 (15.7%)	17 (70.8%)
Delirium type Day 1		
Hyperactive n (%)	9 (8.3%)	9 (37.5%)
Hypoactive n (%)	7 (6.4%)	7 (24%)
Mixed n (%)	1 (0.93%)	1 (4.2%)
Delirium Day 2 n (%)	14 (13%)	13 (57.1%)
Delirium type Day 2		
Hyperactive n (%)	5 (4.6%)	5 (20.8%)
Hypoactive n (%)	8 (7.5%)	8 (33.3%)
Mixed n (%)	1 (0.93%)	1 (4.2%)
Delirium Day 3 n (%)	8 (8%)	8 (33.3%)
Delirium type Day 3		
Hyperactive n (%)	3 (3%)	3 (12.5%)
Hypoactive n (%)	5 (5%)	5 (20.8%)
Mixed n (%)	0	0

Supplementary Table S4. Univariate and multivariate analysis and presentation of different predictive models

Factors	*p-value*	M1 OR	M1 *p*	M2 OR	M2 *p*	M3 OR	M3 *p*	M4 OR	M4 *p*	M5 OR	M5 *p*
Age	**0.002**	1.04 [0.97-1.13]	0.285	**1.07 [1.01-1.15]**	**0.034**	-	-	1.04 [0.92-1.17]	0.526	1.05 [0.96-1.14]	0.304
Sensory deficit	**0.001**	1.75 [0.47-6.47]	0.411	2.08 [0.61-7.13]	0.251	-	-	4.18 [0.60-29.01]	0.148	3.93 [0.75-20.49]	0.104
Benzodiazepine intake	**0.001**	**5.03 [1.38-18.35]**	**0.014**	**5.82 [1.71-19.79]**	**0.004**	-	-	**16.71 [2.45-114.14]**	**0.004**	**7.28 [1.66-31.9]**	**0.007**
Cognitive impairment	**0.001**	**4.4 [1.06-18.5]**	**0.042**	-	-	-	-	**10.77 [1.57-73.84]**	**0.016**	-	-
Polymedication	**0.027**	2.06 [0.67- 6.32]	0.205	1.82 [0.62-5.34]	0.276	-	-	3.46 [0.66-18.13]	0.142	2.16 [0.52-8.93]	0.288
Worst NS in 7 days	**0.004**	1.04 [0.87-1.24]	0.689	-	-	1.12 [0.94-1.34]	0.214	0.78 [0.58-1.05]	0.107	-	-
Duration of hypotension (min)	**0.04**	-	-	-	-	**1.10 [1.01-1.20]**	**0.019**	**1.12 [1.01-1.23]**	**0.029**	**1.10 [0.99-1.21]**	**0.043**
NS D1 morning	**0.004**	-	-	-	-	**1.43 [1.01-2.02]**	**0.037**	**2.34 [1.22-4.48]**	**0.01**	**1.76 [1.1-2.8]**	**0.01**

M = Model ; OR = Odd Ratio

Supplementary Figure S1. Comparison of areas under the curve

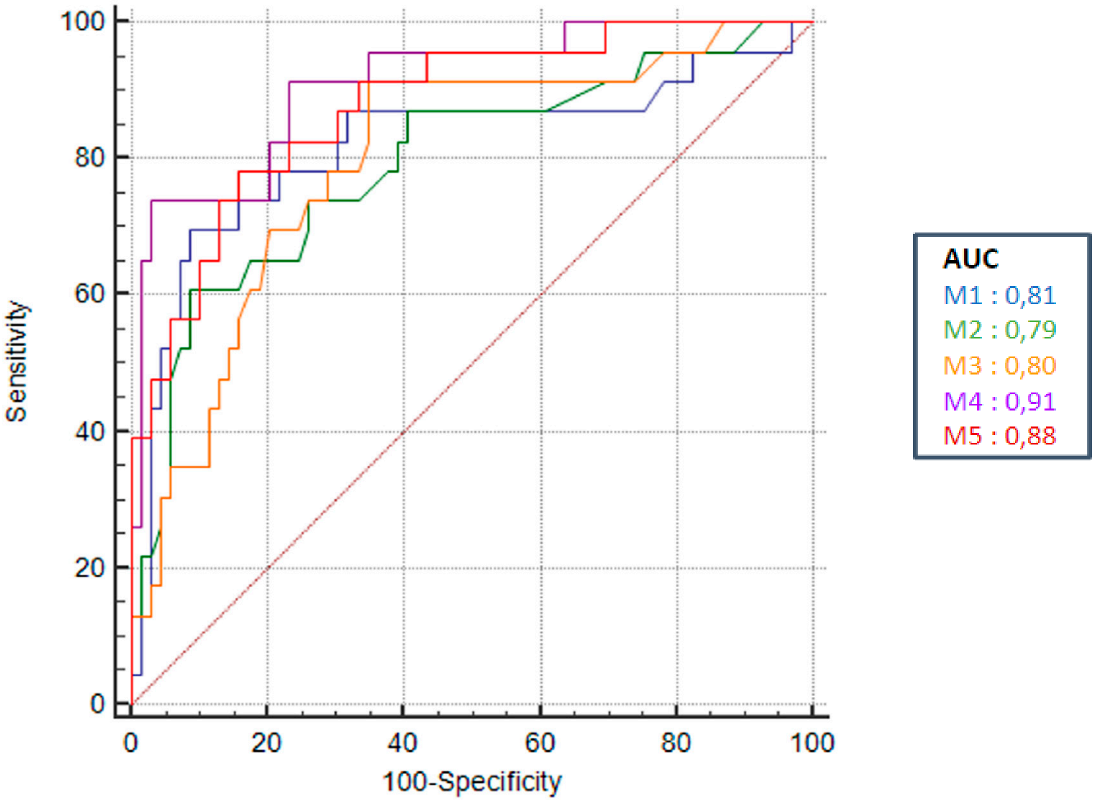

**M1**	-	0.462	0.948	**0.045**	0.086
**M2**	0.462	-	0.798	**0.021**	**0.031**
**M3**	0.948	0.798	-	0.170	0.163
**M4**	**0.045**	**0.021**	0.170	-	0.473
**M5**	0.086	**0.031**	0.163	0.473	-
